# Medical Association—Prize Essays

**Published:** 1855-03

**Authors:** 


### AMERICAN MEDICAL ASSOCIATION.—PRIZE ESSAYS.

At a meeting of the American Medical Association, held in St. Louis, (Mo.,) in May last, a committee was appointed to receive and examine such voluntary communications on subjects connected with medical science as individuals might see fit to make, and to award two prizes of one hundred dollars each to the authors of the two best essays. Notice is hereby given, that all such communications must be sent, post-paid, on or before the first day of April, 1855, to R. La Roche, M. D., Philadelphia. Each communication must be accompanied by a sealed packet, containing the name of the author, which will not be opened unless the accompanying communication be deemed worthy of a prize. Unsuccessful papers will be returned on application to the Committee at any time after the first day of June, 1855; and the successful ones, it is understood, will be published in the *Transactions* of the Association.

Philadelphia, Jan. 1855.

